# Statin Treatment and Clinical Outcomes of Heart Failure Among Africans: An Inverse Probability Treatment Weighted Analysis

**DOI:** 10.1161/JAHA.116.004706

**Published:** 2017-04-01

**Authors:** Kwadwo Osei Bonsu, Isaac Kofi Owusu, Kwame Ohene Buabeng, Daniel D. Reidpath, Amudha Kadirvelu

**Affiliations:** ^1^ School of Medicine and Health Sciences Monash University Bandar Sunway Malaysia; ^2^ Accident and Emergency Directorate Komfo Anokye Teaching Hospital Kumasi Ghana; ^3^ Directorate of Medicine Komfo Anokye Teaching Hospital Kumasi Ghana; ^4^ Department of Medicine School of Medical Sciences College of Health Sciences Kwame Nkrumah University of Science and Technology Kumasi Ghana; ^5^ Department of Pharmacy Practice College of Health Sciences Kwame Nkrumah University of Science and Technology Kumasi Ghana

**Keywords:** Africans, inverse probability treatment weighting, outcome, race and ethnicity, statin therapy, Heart Failure, Clinical Studies, Epidemiology, Race and Ethnicity, Treatment

## Abstract

**Background:**

Randomized control trials of statins have not demonstrated significant benefits in outcomes of heart failure (HF). However, randomized control trials may not always be generalizable. The aim was to determine whether statin and statin type–lipophilic or –hydrophilic improve long‐term outcomes in Africans with HF.

**Methods and Results:**

This was a retrospective longitudinal study of HF patients aged ≥18 years hospitalized at a tertiary healthcare center between January 1, 2009 and December 31, 2013 in Ghana. Patients were eligible if they were discharged from first admission for HF (index admission) and followed up to time of all‐cause, cardiovascular, and HF mortality or end of study. Multivariable time‐dependent Cox model and inverse‐probability‐of‐treatment weighting of marginal structural model were used to estimate associations between statin treatment and outcomes. Adjusted hazard ratios were also estimated for lipophilic and hydrophilic statin compared with no statin use. The study included 1488 patients (mean age 60.3±14.2 years) with 9306 person‐years of observation. Using the time‐dependent Cox model, the 5‐year adjusted hazard ratios with 95% CI for statin treatment on all‐cause, cardiovascular, and HF mortality were 0.68 (0.55–0.83), 0.67 (0.54–0.82), and 0.63 (0.51–0.79), respectively. Use of inverse‐probability‐of‐treatment weighting resulted in estimates of 0.79 (0.65–0.96), 0.77 (0.63–0.96), and 0.77 (0.61–0.95) for statin treatment on all‐cause, cardiovascular, and HF mortality, respectively, compared with no statin use.

**Conclusions:**

Among Africans with HF, statin treatment was associated with significant reduction in mortality.

## Introduction

Heart failure (HF) has become a major public health and clinical priority worldwide.^1^ With the world's aging population coupled with epidemiological transition, the burden of HF is expected to rise in the foreseeable future.^1^, ^2^ Statins are well known to reduce cardiovascular events,^3^, ^4^ and remain key to preventing HF. More recently there have been some discussions about potential benefit of statins for treatment of established HF because of various pleiotropic (ie, non‐cholesterol‐lowering) effects.^5^

Notwithstanding the benefit of statin therapy in prevention, the evidence for the role of statins in the treatment of established HF remains unclear. A number of nonrandomized studies and post hoc analyses of randomized control trials (RCTs) evaluating treatments other than statins suggest that statin therapy improves clinical outcomes in patients with HF,^6^, ^7^ and benefits were also shown in several small RCTs identifying improved surrogate and mortality outcomes in HF.^8^, ^9^, ^10^ In contrast, large RCTs—the Controlled Rosuvastatin Multinational Study in Heart Failure (CORONA) study^11^ and Gruppo Italiano per lo Studio della Sopravvivenza nell'Insufficienza cardiaca (GISSI‐HF)^12^—which were undertaken to evaluate these promising findings, did not show significant outcome benefits in primary end points compared with placebo. The result from the CORONA trial, however, did show significant reductions in hospitalizations and improved survival in subgroups of patients with low galectin‐3^13^ and N‐terminal pro‐B‐type natriuretic peptide^14^ levels on rosuvastatin therapy.

It is noteworthy that CORONA and GISSI‐HF evaluated the same drug (rosuvastatin) and dose (10 mg). Two subsequent meta‐analyses of RCTs^15^, ^16^ that included the CORONA and GISSI‐HF trials found no significant outcome benefit of statin therapy versus placebo in HF. A closer examination of the management of RCTs included in the meta‐analyses shows that the researchers treated the statins as if they were a uniform class of drugs, which they are not, and thus failed to compare the effects of statin types utilized in each trial. Within the statins, there are 2 different types, which can be identified by their hydrophilicity or lipophilicity. Rosuvastatin (hydrophilic statin) evaluated in CORONA and GISSI‐HF is suggested to have played a critical part in the neutral outcome in both large RCTs and possibly skewed previous meta‐analyses toward the results of these 2 large trials.

Subsequent to CORONA and GISSI‐HF, several studies demonstrated improved clinical outcomes with statin therapy in HF.^17^, ^18^, ^19^ These studies were nonrandomized, but did suggest that lipophilic statins may have better outcomes than hydrophilic statins in treatment of HF.^5^ Lipophilic statins have also shown improved surrogate outcomes (cardiac function and inflammation) and significant reductions in hospitalizations for worsening HF, all‐cause, and cardiovascular mortality compared with hydrophilic statin treatment in indirect comparison meta‐analyses of RCTs.^20^, ^21^

In addition to the doubt raised in the generalizability of the earlier large statin trials, because of the focus on hydrophilic statins, the patient groups were overwhelmingly of white background. Racial and ethnic differences play an important role in patient characteristics, treatment, and prognoses of HF.^22^, ^23^, ^24^, ^25^ In particular, blacks compared with other racial groups, are at increased risk of developing HF.^23^, ^24^ Re‐analysis of major HF clinical trials data suggests that black patients have varied responses to approved treatments for HF compared with whites.^26^, ^27^, ^28^, ^29^ In this present study, we evaluate the association between statin use, statin type, and long‐term outcomes of an African population with HF.

## Method

The study design was a retrospective longitudinal cohort of newly diagnosed HF patients aged ≥18 years hospitalized between January 1, 2009 and December 31, 2013. HF was diagnosed using the modified Framingham criteria^30^, ^31^ and echocardiographic data. Patients were eligible for the study if they were hospitalized for HF as a primary cause of admission or HF was diagnosed during hospitalization, when HF was not the initial reason for admission. The first admission for HF was considered the index admission. The follow‐up commenced from the date of discharge of the index admission to the time of all‐cause, cardiovascular, or worsening HF mortality; loss to follow‐up; or the end of the study.

### Study Site

The study was conducted at the cardiac clinic, Directorate of Medicine, Komfo Anokye Teaching Hospital, Kumasi, Ghana. Komfo Anokye Teaching Hospital is a 1200‐bed hospital located in Kumasi, which is the regional capital of the Ashanti region of Ghana and the only tertiary facility with a cardiac clinic that serves patients from the northern half of Ghana. Approximately 85% of patients who seek health care at Komfo Anokye Teaching Hospital subscribe to the National Health Insurance Scheme.

The study was approved by the Committee on Human Research, Publications and Ethics of Kwame Nkrumah University of Science and Technology, Ghana and the Monash University Human Research Ethics Committee, Australia. Patient consent was not required.

### Data Source

Data for the study were abstracted from clinical records of HF patients from the cardiac clinic, Directorate of Medicine of Komfo Anokye Teaching Hospital. Data were sourced from patients' medical records linked to claims records from the pharmacy department and the National Health Insurance Scheme unit of the hospital. The medical records contain physical signs, diagnosis, laboratory investigations and results, chest radiograph reports, echocardiography reports, electrocardiograph and procedures conducted on patients, prescribed medications and cost covered during admission or hospital visit linked with the Ghana Diagnosis‐Related Group codes. The records contain medical and drug history as well as demographic data of patients. The demographic data include age, sex, height, weight, marital status, and highest level of education of patients. Also included in the data are admission dates, discharge dates, as well as death dates if patients died.

### Exposure

The exposure of interest was statin prescription at discharge from index admission or during clinic visit. The comparison was no statin prescription on optimal treatment at discharge from admission or during clinic visit. We defined statin treatment eligibility as both ischemic and nonischemic etiology of HF, no documented contraindication, no allergies to statins, and without prior exposure to statin at least 3 months before index admission for HF.^32^ We employed the new user approach to avoid bias introduced by the inclusion of prevalent statin users into the study cohort. Thus, we required that patients were naive to statin therapy at index admission to merit inclusion. A patient's exposure to statin treatment was assessed for the study period (January 1, 2009–December 31, 2013). Exposure was classified on a monthly basis by assessment of the days' supply of filled prescriptions. Every person‐month during study follow‐up was classified according to the use of statins. All prescriptions for statins were retrieved, and the length of each prescription was calculated based on the recorded number of tablets prescribed and dispensed and the daily dose; where these data were not available, the median value (28 days) was assumed. Statin use in a month was defined as use when the days' supply for statins covered 15 or more days in that month. No statin use in a month was defined as no prescription for statin or days' supply covered less than 15 days in that month or any preceding months.^33^, ^34^ Thus statin users could become nonusers of statin during follow‐up. Exposure to statin treatment was therefore time varying or time dependent and could change over the course of follow‐up.

### Outcome Assessment

The study outcomes were time to all‐cause, cardiovascular, and worsening HF mortality following discharge from the index admission. Cardiovascular and HF mortality were defined based on autopsy reports, clinical notes, and Ghana Diagnosis‐Related Group codes in claim records.

### Censoring

Patients who did not experience any of the study end points or outcomes were censored. The censoring dates were defined as (1) the end of study period or (2) the date on which the patient's data were no longer available.

### Covariates

The variation in survival rates may be associated with time‐independent demographic and time‐dependent and ‐independent clinical and treatment factors accounting for 33 covariates for analysis. Demographic factors included age, sex, BMI, history of cigarette smoking, and highest level of education. The time‐independent clinical factors were medical history/comorbidities, which included HF etiology, anemia, atrial fibrillation, chronic liver disease, chronic obstructive pulmonary disease, prior myocardial infarction, coronary artery disease, prior angina pectoris, diabetes mellitus, hypertension, stroke, dilated cardiomyopathy, and chronic kidney disease. The time‐dependent clinical factors were physical signs (heart rate, New York Heart Association [NHYA] functional class, diastolic blood pressure, and systolic blood pressure) and results of investigations (ejection fraction, low‐density lipoprotein‐cholesterol [LDL‐C] and high‐density lipoprotein‐cholesterol [HDL‐C]) conducted during admissions and/or scheduled visits. Treatment factors included prescribed co‐medications at discharge or scheduled visit (angiotensin‐converting enzyme inhibitors/angiotensin II receptor blockers, aldosterone antagonist, β‐blockers, digoxin, diuretics, calcium channel blockers, oral anticoagulants, and nitrates).

### Statistical Analysis

Data analyses were performed using R statistical software version 3.2.4 (R foundation for Statistical Computing, Vienna, Austria). We used χ^2^ and *t* test to examine bivariate associations between predictor variables and outcomes for categorical and continuous variables, respectively. Two different approaches were used to examine the treatment effect of statin on mortality outcomes of HF. First, a time‐dependent Cox model was developed, and second, a marginal structural Cox model using inverse probability weights was constructed.^33^, ^35^ Missing data for variables were handled by multiple imputation approach based on the pattern for all available observations. For all analyses, a level of significance was set to 0.05 and all reported *P* values are 2‐sided.

### Time‐Dependent Cox Model

Crude mortality rates for statin treatment versus no statin use were compared. We used the Kaplan–Meier method to estimate unadjusted mortality by statin treatment versus no statin use, and the log‐rank test was used to compare the groups. Next, multivariable time‐dependent Cox models of time to mortality outcomes were constructed. The independent variables used in the Cox regression were 33 covariates comprising time‐independent demographic and clinical factors as well as time‐dependent clinical and treatment factors updated periodically during follow‐up. Patients were censored if they did not reach the outcome until December 31, 2013 (end of the study) or last date patient records were traceable before end of study. Hazards ratios were obtained from the model after adjusting for the covariates mentioned above. LDL‐C levels reported during follow‐up may be time‐dependent confounder in the present study. It is an intermediate variable affected by previous treatment and predicting future treatment and an independent risk factor for adverse outcomes in HF. Thus, simply adding this variable in the time‐dependent Cox model may introduce bias and cannot provide causal effect of statin treatment on outcomes in HF.^36^

### Marginal Structural Cox Model

To estimate the causal effect of statin versus no statin use on mortality outcomes in the presence of time‐varying confounding factors, marginal structural Cox model using inverse‐probability‐treatment‐weighting (IPTW) was employed. The IPTW approach creates a pseudopopulation of original subjects who account for themselves and for subjects with similar characteristics who received the alternate exposure.^33^, ^35^ With time‐independent exposure, IPTW creates a pseudopopulation in which all subjects are considered conditionally exchangeable by achieving a balance between the treated and nontreated groups on the baseline covariates at the start of the study.^33^, ^37^ Unlike time‐independent exposures, longitudinal studies with time‐varying treatment employ marginal structural models (MSMs) using the IPTW, which is updated at various time points to achieve balance between the groups not only at baseline but also at different time points. Thus, MSM allows for the control of time‐dependent confounders that predict the subsequent treatment and are predicted by previous treatment.^37^ MSMs using IPTW are related to propensity scoring.^38^, ^39^ The IPTW approach has been developed to utilize all sample information with assigned weights by making an unbiased estimation of the true risk difference with the lowest standard error of the estimated risk difference, the lowest mean‐squared error, and approximately correct type I error rates.^40^, ^41^ It has also been shown to handle longitudinal data characterized by time‐varying treatments and covariates better than conventional propensity score methods.^40^, ^42^ Using the same 33 covariates for the time‐dependent Cox model, case‐weight estimation was done to predict the inverse probability weight for statin use and censoring.^40^, ^43^ A large variability in propensity score distribution plausibly attributable to high correlations of some covariates with treatment means treatment patterns will have extremely large weights.^37^ Thus, we used an approach proposed by Robins et al^44^ and Hernan et al^39^ that recommends replacing the IPTW with stabilized weights to reduce this variability and ensure that estimated treatment effect remains unbiased.^37^ These stabilized weights were estimated from the product of treatment and censoring weights.

To estimate the stabilized weights for use in MSM, first, we created treatment history weights at various time intervals. We calculated the treatment history weights for each time interval as conditional probability of receiving the observed treatment based on the treatment history (treatment in prior time interval) and the baseline covariates divided by conditional probability of receiving the observed treatment based on the treatment history and the baseline covariates as well as the time‐dependent covariates (LDL‐C, HDL‐C, and left ventricular ejection fraction).^39^, ^45^ Second, the censoring history weight to adjust for censoring by loss to follow‐up or end of study was calculated by an approach similar to the estimation of treatment history weights. The 2 calculated weights (treatment and censoring) were multiplied to create stabilized weights for each subject in each period.^33^ Finally, to estimate the treatment effect of statin on observed mortality outcomes, we constructed a weighted Cox regression model with robust standard errors estimation, treating each person‐period as an observation. This modeling approach assumes no unmeasured confounding, correct model specification, and positivity; thus, treatment effects estimated in this model have causal interpretation.^45^ We compared the estimates (hazard ratios with corresponding 95% CI) obtained from MSM with that obtained from the time‐dependent Cox model to ascertain bias because of time‐varying confounding factors. In our entire Cox modeling, the proportional hazards assumption was tested by scaled Schoenfeld residuals, and the presence of extreme outliers was assessed by dfbetas. No violations to the proportional hazards assumption or possible influential outliers were found.

### Subgroup and Interaction Analyses

Several planned subgroup analyses were carried out to test the robustness of the study findings. First, we carried out an analysis of the study population restricted to those who were prescribed lipophilic statins versus no statin prescription on optimal treatment for HF, and a comparable analysis of those prescribed hydrophilic statins. Finally, we compared the treatment effect of lipophilic versus hydrophilic statins on mortality outcomes. Interactions between statin treatment and clinically relevant variables were examined using MSMs with IPTW analysis for all‐cause mortality. Continuous variables were categorized for easier visual interpretation.

## Results

### Patient Characteristics

**Figure 1:**
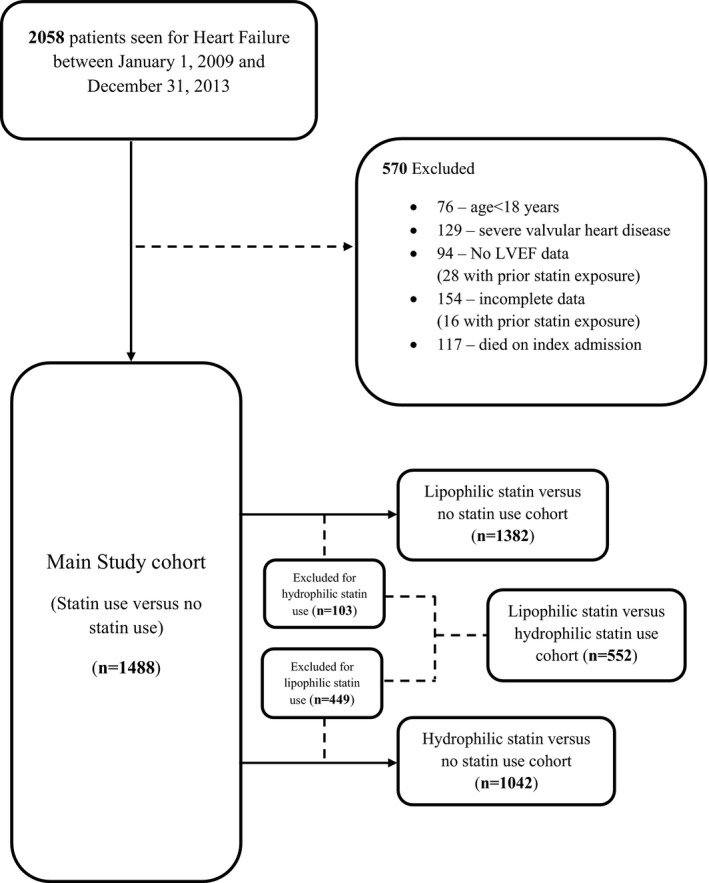
Flow chart showing the derivation of various study cohorts for data analyses. LVEF indicates left ventricular ejection fraction.

There were 1488 HF patients included in the retrospective cohort. Figure 1 is a flow chart illustrating how various study cohorts were derived for data analyses. The mean (±SD) age of our cohort was 60.3 (±14.2) years and ≈54% of the patients were women. Almost 5% of the patients reported to have ever smoked. Of these patients, 552 (37.2%) received statins and 939 (62.8%) did not receive statin treatment on optimal treatment for HF. Table 1 provides the baseline characteristics of the study cohort with and without statin treatment.

**Table 1:** Demographics and Clinical Characteristics of Overall Cohort and Statin Use

	Overall (n=1488)	Statin Use (n=552)	No Statin Use (n=936)	*P* Value^a^
Demographics				
Sex female, %	54.4	52.7	55.3	0.353
Mean age (SD), years	60.3 (14.2)	61.0 (14.2)	59.9 (14.2)	0.132
Age group, %				
<65	62.8	60.3	64.3	0.138
≥65	37.2	39.7	35.7	…
Education, %				
Basic education	20.4	20.3	20.5	0.401
Secondary education	47.7	45.3	49.1	…
Tertiary education	30.6	33.0	29.2	…
No formal education	1.3	1.4	1.2	…
Clinical characteristics				
NYHA, %				
I	11.1	11.8	10.7	0.003
II	46.4	49.3	44.7	…
III	29.8	29.9	29.8	…
IV	12.7	9.1	14.9	…
Nonischemic etiology, %	89.6	86.1	91.7	0.001
Readmission, %	45.2	42.9	46.6	0.627
Ever smoked, %	4.6	4.3	4.7	0.852
Chronic liver disease, %	0.6	0.7	0.5	0.911
Chronic kidney disease, %	17.6	20.8	15.7	0.015
Anemia, %	9.9	12.3	8.4	0.020
Prior myocardial infarction, %	4.0	4.5	3.6	0.472
Chronic obstructive pulmonary disease, %	2.2	2.2	2.2	1.000
Stroke, %	5.8	6.9	5.2	0.232
Hypertension, %	61.2	64.1	59.4	0.080
Diabetes mellitus, %	22.8	24.8	21.7	0.185
Dilated cardiomyopathy, %	19.9	17.9	21.0	0.166
Prior angina pectoris, %	2.7	3.8	2.0	0.060
Prior coronary artery disease, %	10.2	12.7	8.8	0.020
Atrial fibrillation, %	20.7	17.3	23.0	0.009
Systolic blood pressure (SD), mm Hg	134.4 (25.6)	135.5 (25.7)	133.7 (25.6)	0.200
Diastolic blood pressure (SD), mm Hg	85.1 (14.7)	85.3 (13.7)	85.0 (15.3)	0.759
LDL‐C (SD), mmol/L	3.9 (2.4)	4.5 (2.1)	3.5 (2.6)	<0.001
HDL‐C (SD), mmol/L	1.6 (1.5)	1.7 (1.5)	1.5 (1.4)	0.019
Heart rate (SD), beat per minute	70.8 (29.6)	67.9 (29.7)	72.4 (29.5)	0.005
BMI (SD), kg/m^2^	25.4 (12.0)	24.6 (11.9)	25.9 (12.1)	0.040
Ejection fraction % (SD)	52.9 (16.4)	54.2 (17.1)	52.2 (15.9)	0.024
Ejection fraction by group, %				
<50	41.1	38.4	42.6	0.122
≥50	58.9	61.6	57.4	…
Discharge medications				
ACE inhibitor/ARB, %	62.0	61.2	62.4	0.696
Aldosterone antagonist, %	28.0	28.3	27.8	0.888
Digoxin, %	16.3	17.4	15.6	0.405
Diuretic, %	68.4	67.6	68.9	0.632
β‐Blocker, %	32.5	32.1	32.8	0.814
Oral anticoagulant, %	0.9	2.0	0.3	0.003
Nitrate, %	2.1	2.7	1.7	0.260
Calcium antagonist, %	44.9	50.4	41.7	0.001

Mean and SD are reported for continuous data and percentages for categorical data. ACE indicates angiotensin‐converting enzyme; ARB, angiotensin receptor blocker; HDL‐C, high‐density lipoprotein‐cholesterol; LDL‐C, low‐density lipoprotein‐cholesterol; NYHA, New York Heart Association.

a Comparing statin use and no statin use.

### Clinical Factors

Our cohort had a diverse mix of patients with differences in HF severity as measured by ejection fraction and NYHA functional class. The mean (±SD) ejection fraction of patients in the overall cohort was 52.9 (±16.4). This differed between statin and nonstatin users at baseline (54.2 [±17.1] versus 52.2 [±15.9], *P*=0.024). About 76.2% of the overall cohort was in NYHA class II and III, 11.1% belonged to class I, and 12.7% were in class IV at baseline. Statin and nonstatin users had significant differences across the NYHA classes (*P*=0.003). Statin users were symptomatic of HF (NYHA II and III) but nonstatin users had a significantly higher proportion of HF patients with severe symptoms (NYHA IV). In our study cohort, HF was predominantly of nonischemic etiology with only about 10% of ischemic etiology of HF. We found significant differences between statin users and nonstatin users regarding clinical factors such as HDL‐C, LDL‐C, and heart rate. Statin users had significantly lower heart rate and HDL‐C levels, but higher LDL‐C levels compared with nonstatin users. Generally, the patients in the overall cohort had considerable comorbidity burden, with over 60% of the cohort having at least 1 comorbid condition. Hypertension was the most frequent comorbid condition with 61.2% of the patients but did not differ between statin and nonstatin users. Diabetes mellitus (22.8%), atrial fibrillation (20.7%), idiopathic dilated cardiomyopathy (19.9%), chronic kidney disease (17.6%), coronary artery disease (10.7%), and anemia (9.9%) were some of the reported comorbidities. Statin and nonstatin users differed significantly for the presence of atrial fibrillation (*P*=0.009), chronic kidney disease (*P*=0.015), coronary artery disease (*P*=0.020), and anemia (*P*=0.020).

### Medication Utilization

In our cohort of 1488 HF patients, 37% received statin treatments during follow‐up. Of the patients who were prescribed statins, 18.7% received rosuvastatin (5–20 mg) as the only hydrophilic statin, whereas the remaining were lipophilic statins comprising atorvastatin (10–80 mg) (49.3%), fluvastatin (20–80 mg) (21.2%), and simvastatin (20–80 mg) (11.1%). Patients in our cohort showed frequent utilization of other medication classes for treatment of HF. Among the co‐medications, diuretics were prescribed for the majority of the patients (68.4%), followed by angiotensin‐converting enzyme inhibitor/angiotensin II receptor blockers (62.0%), calcium channel antagonists (44.9%), β‐blockers (32.5%), and aldosterone antagonists (28.0%). The diuretic use implies prescription for thiazide and loop diuretics. Utilization of all co‐medications was similar except for the use of calcium channel antagonists (*P*=0.001) and oral anticoagulants (*P*=0.003), which significantly differed between statin and nonstatin users.

### Statin Treatment on Mortality Outcomes

Patients in the overall cohort had 9306 person‐years of observation for the period of study. During follow‐up, 472 (31.7%) deaths occurred, 249 among women and 223 among men. Among patients who were prescribed statins, 166 deaths occurred (30.1% of statin patients), whereas 306 (32.6%) died in the nonstatin group. The median survival after discharge from index admission for HF is 3.26, 4.13, and 2.97 years for overall cohort, statin users, and nonstatin users, respectively. We found survival rates for the overall cohort at 1, 3, and 5 years to be 90.3%, 64.7%, and 38.4%, respectively. The survival rates differed between statin and nonstatin users. For statin users, 1‐, 3‐, and 5‐year survival rates were 90.4% (95% CI 87.9–93.0%), 59.9% (95% CI 54.6–65.7%), and 44.0% (95% CI 36.8–52.7%), whereas corresponding rates among nonstatin users were 90.2% (95% CI 88.3–92.2%), 49.5% (95% CI 44.8–54.7%), and 32.6% (95% CI 26.7–39.8%), respectively. The crude 5‐year all‐cause mortality was 11.2% in patients treated with statins and 20.6% for those without statins (*P*=0.065). Cardiovascular causes accounted for mortality of 157 (10.5%) of patients who received statins and 297 (19.9%) of nonstatin users during follow‐up (*P*=0.039). Worsening HF accounted for 5‐year crude mortality of 146 (9.8%) among statin users and 286 (19.2%) in nonstatin users (*P*=0.019).

### Time‐Dependent Cox Model

The unadjusted hazard ratios for statin treatment on outcomes throughout follow‐up were 0.77 (95% CI 0.63–0.93, *P*=0.006) for all‐cause mortality, 0.75 (95% CI 0.62–0.91, *P*=0.004) for cardiovascular mortality, and 0.73 (95% CI 0.59–0.89, *P*=0.003) for worsening HF mortality. After adjustment for age and sex, the effects of statin treatment persisted on all‐cause mortality; 0.75 (95% CI 0.62–0.91, *P*=0.003), cardiovascular mortality; 0.74 (95% CI 0.61–0.89, *P*=0.002), and worsening HF mortality; 0.72 (95% CI 0.59–0.88, *P*=0.001). The overall multivariable time‐dependent Cox model adjusting for age, sex, and clinical and treatment factors further found that, compared with nonstatin users, statin use was associated with significant reduction in all‐cause mortality; 0.68 (95% CI 0.55–0.83, *P*=0.0002), cardiovascular mortality; 0.67 (95% CI 0.54–0.82, *P*=0.0001), and worsening heart failure mortality; 0.63 (95% CI 0.51–0.79, *P*<0.0001).

### Marginal Structural Cox Model

**Figure 2:**
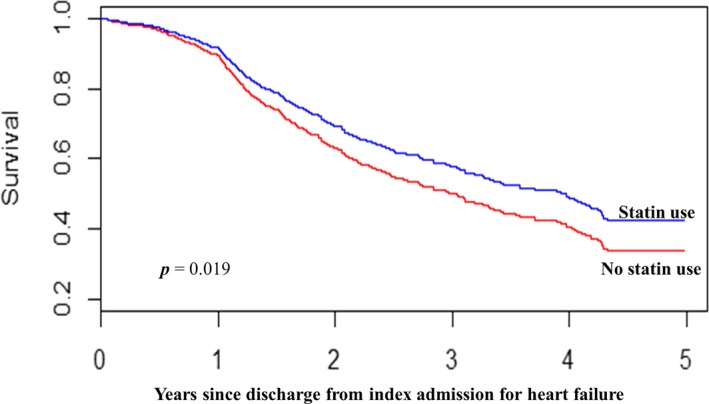
Kaplan–Meier survival curves for statin vs no statin treatment in the inverse‐probability‐treatment‐weighted population.

The stabilized MSM weights had symmetric distribution with mean (±SD) of 0.997 (±0.285), which was centered around the ideal value of 1 at all times during follow‐up. After adjusting for the time‐dependent confounders in addition to other confounders (listed in Table 1) and applying stabilized weights to Cox regression model, statin use was associated with statistically significant reduction in all‐cause mortality; 0.79 (95% CI 0.65–0.96, *P=*0.019) cardiovascular mortality; 0.77 (95% CI 0.63–0.95, *P*=0.013) and worsening HF mortality; 0.76 (95% CI 0.62–0.95, *P*=0.013) compared with those who did not receive statin treatment. Figure 2 illustrates the adjusted Kaplan–Meier survival curves for statin versus no statin treatment in all‐cause mortality for the inverse probability weighted population. These estimates were similar to the hazard ratios obtained from the time‐dependent Cox model (Table 2).

**Table 2:** Association Between Statin Use and Outcomes With Time‐Dependent Cox and Marginal Structural Cox Model With IPTW Estimation

Outcomes/Model	Number of Events	Number Censored	5‐Year Hazard Ratios	95% CI	*P* Value
Analysis of all patients on statin vs no statin treatment					
All‐cause mortality	472	1016			
			Time‐dependent Cox model		
Unadjusted			0.77	0.63 to 0.93	0.006
Age and sex adjusted			0.75	0.62 to 0.91	0.003
Overall			0.68	0.55 to 0.83	<0.001
			MSM with IPTW		
			0.79	0.65 to 0.96	0.019
Cardiovascular mortality	454	1034			
			Time‐dependent Cox model		
Unadjusted			0.75	0.62 to 0.91	0.004
Age and sex adjusted			0.74	0.61 to 0.89	0.002
Overall			0.67	0.54 to 0.82	<0.001
			MSM with IPTW		
			0.77	063 to 0.95	0.013
Heart failure mortality	432	1056			
			Time‐dependent Cox model		
Unadjusted			0.73	0.60 to 0.90	0.003
Age and sex adjusted			0.72	0.59 to 0.88	0.001
Overall			0.63	0.51 to 0.79	<0.001
			MSM with IPTW		
			0.77	0.61 to 0.95	0.013
Analysis restricted to patients who received lipophilic statins vs no statin treatment					
All‐cause mortality	447	938			
			Time‐dependent Cox model		
Unadjusted			0.77	0.63 to 0.94	0.009
Age and sex adjusted			0.75	0.61 to 0.92	0.006
Overall			0.68	0.54 to 0.84	<0.001
			MSM with IPTW		
			0.79	0.64 to 0.97	0.024
Cardiovascular mortality	431	954			
			Time‐dependent Cox model		
Unadjusted			0.76	0.62 to 0.93	0.008
Age and sex adjusted			0.74	0.60 to 0.91	0.004
Overall			0.67	0.53 to 0.83	<0.001
			MSM with IPTW		
			0.77	0.62 to 0.96	0.018
Heart failure mortality	410	975			
			Time‐dependent Cox model		
Unadjusted			0.74	0.59 to 0.91	0.005
Age and sex adjusted			0.72	0.58 to 0.90	0.003
Overall			0.62	0.49 to 0.78	<0.001
			MSM with IPTW		
			0.77	0.61 to 0.96	0.020
Analysis restricted to patients who received hydrophilic statins vs no statin treatment					
All‐cause mortality	331	708			
			Time‐dependent Cox model		
Unadjusted			0.76	0.51 to 1.13	0.168
Age and sex adjusted			0.73	0.48 to 1.09	0.129
Overall			0.71	0.47 to 1.07	0.101
			MSM with IPTW		
			0.82	0.54 to 1.23	0.333
Cardiovascular mortality	330	709			
			Time‐dependent Cox model		
Unadjusted			0.72	0.47 to 1.09	0.125
Age and sex adjusted			0.70	0.45 to 1.07	0.098
Overall			0.69	0.44 to 1.07	0.099
			MSM with IPTW		
			0.78	0.50 to 1.19	0.256
Heart failure mortality	308	731			
			Time‐dependent Cox model		
Unadjusted			0.72	0.47 to 1.10	0.130
Age and sex adjusted			0.7	0.45 to 1.08	0.105
Overall			0.68	0.43 to 1.08	0.098
			MSM with IPTW		
			0.76	0.49 to 1.19	0.230
Comparative analysis restricted to patients who received lipophilic vs hydrophilic statins					
All‐cause mortality	166	386			
			Time‐dependent Cox model		
Unadjusted			0.98	0.65 to 1.47	0.915
Age and sex adjusted			0.99	0.65 to 1.51	0.969
Overall			1.02	0.65 to 1.54	0.990
			MSM with IPTW		
			1.02	0.61 to 1.65	0.994
Cardiovascular mortality	157	395			
			Time‐dependent Cox model		
Unadjusted			0.94	0.61 to 1.45	0.790
Age and sex adjusted			0.96	0.61 to 1.47	0.803
Overall			0.95	0.61 to 1.50	0.838
			MSM with IPTW		
			0.95	0.56 to 1.61	0.848
Heart failure mortality	146	406			
			Time‐dependent Cox model		
Unadjusted			0.96	0.62 to 1.50	0.870
Age and sex adjusted			0.96	0.62 to 1.50	0.865
Overall			1.00	064 to 1.58	0.994
			MSM with IPTW		
			0.99	0.59 to 1.70	0.993

Overall model is age‐ and sex‐adjusted model+time‐dependent and time‐independent clinical and treatment factors. IPTW indicates inverse probability treatment weight; MSM, marginal structural (Cox) model.

The treatment effect of statins on mortality outcomes persisted after adjustment for age and sex, time‐dependent and time‐independent clinical and treatment factors in both Cox multivariate and MSM with IPTW analyses of the study cohort.

### Subgroup and Interaction Analyses

**Figure 3:**
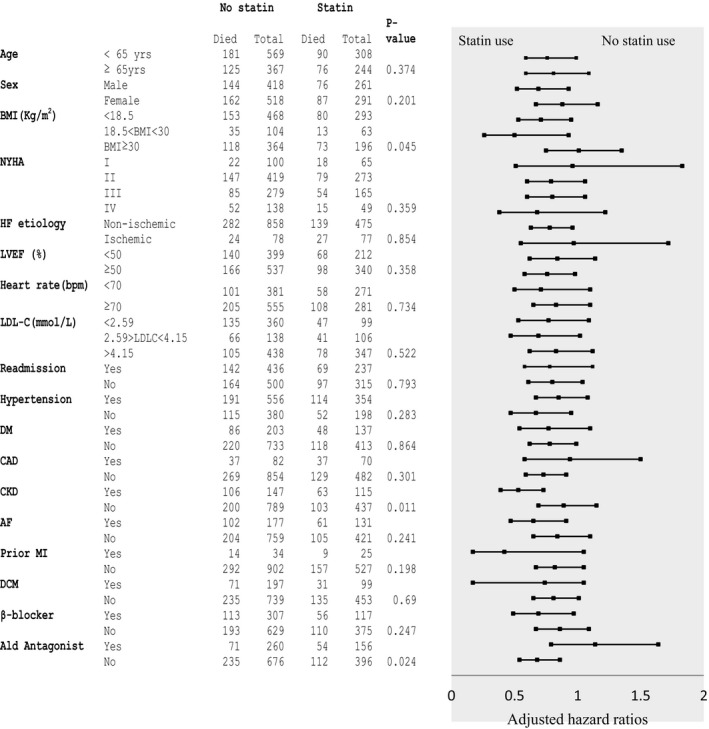
Forest plot illustrating hazard ratios for all‐cause mortality associated with statin use in predefined subgroups in the inverse‐probability‐treatment‐weighted population after adjustment for interaction between statin use and clinically relevant variables. Note squares represents hazard ratio and lines represent the associated 95% CI. Continuous variables were analyzed as categorical variables at clinically relevant cutoffs for display in this figure. AF indicates atrial fibrillation; Ald, aldosterone antagonist; CAD, coronary artery disease; CKD, chronic kidney disease; DCM, dilated cardiomyopathy; DM, diabetes mellitus; HF, heart failure; LDL‐C, low‐density lipoprotein cholesterol; LVEF, left ventricular ejection fraction; MI, myocardial infarction; NYHA, New York Heart Association functional class; *P*‐value, *P*‐value for interaction.

Table 2 shows the treatment effect of statins in several subgroup analyses carried out on the study population. Findings from the analysis of data from patients who were prescribed lipophilic statins compared with those who did not receive statin treatment were essentially identical in magnitude and significance to those of the primary analysis for both time‐dependent Cox and MSM with IPTW models. In the analyses restricted to patients who received hydrophilic statins, there was no significant reduction in mortality outcomes compared with those who did not receive statin treatment. Figure 3 shows a forest plot illustrating the association between statin use and all‐cause mortality in subgroups with adjustment for interactions between clinically relevant variables and statin treatment. There were no significant interactions between most of the clinically relevant variables and statin treatment except for BMI (*P*=0.045), chronic kidney disease (*P*=0.011), and aldosterone antagonist use (*P*=0.023).

## Discussion

In our cohort of black Africans with newly diagnosed HF, use of statin was associated with reductions in mortality for all‐cause, cardiovascular, and worsening HF mortality. The findings were consistent across both the IPT‐weighted analysis and the overall analysis adjusting for clinically relevant covariates. Importantly, lipophilic statin use in patients with HF was associated with reduced mortality outcomes (compared with no statin treatment), but this effect was not observed in patients using hydrophilic statins in HF.

These findings are in contrast to the CORONA^11^ and GISSI‐HF^12^ trials, although consistent with others.^6^, ^7^, ^8^, ^9^, ^10^, ^17^, ^18^, ^19^ Our observational cohort was relatively younger and had a considerable proportion of mild HF (mean age 60 years and <30% in NYHA class III), whereas CORONA had elderly with advanced HF (mean age 73 years and >60% in NYHA class III). While significant benefit from statin therapy is typically seen in patients with ischemic heart disease,^3^ at some point after development of HF, their disease becomes too advanced to be modified by statin therapy,^46^ and this could be the case in CORONA. In addition, it has been suggested that statin treatment can modify coronary events, which is an important driver of outcomes in milder HF, but may not substantially improve progressive loss of pump function in advanced HF, especially when administered on top of optimal treatment for HF.^47^ Indeed, post hoc analysis of the CORONA trial data did show significant reduction in primary end point from rosuvastatin therapy in patients with the lowest N‐terminal pro‐B‐type natriuretic peptide tertile.^14^ In the analysis, patients with plasma concentrations in the lowest tertile of N‐terminal pro‐B‐type natriuretic peptide were younger and had mild symptoms of HF. Although we could not perform similar analysis because of lack of N‐terminal pro‐B‐type natriuretic peptide data, the large proportion of patients with mild HF coupled with our relatively younger cohort could plausibly explain the observed reduction in mortality outcomes seen in the present study.

The findings of this study are consistent with recent observational studies with mixed patient population of ischemic and nonischemic HF.^17^, ^18^, ^19^, ^48^, ^49^ Although our cohort is mixed, it may be considered predominantly a nonischemic HF population, as only ≈10% had ischemic HF. The effects of statin therapy in nonischemic HF patients have been investigated in previous studies.^9^, ^50^ Despite the small sample sizes (42–108 patients) and relatively shorter follow‐up (6–12 months), these trials found that statin therapy significantly improved various biomarkers, including left ventricular ejection fraction, endothelial function, and serum inflammatory markers such as C‐reactive protein, interleukin‐6, and tumor necrosis factor‐α.^9^, ^50^ These pleiotropic effects are suggested to be the mechanisms underlying the benefits of statins seen in patients with nonischemic HF.^5^, ^51^ Evidence from recent meta‐analyses of RCTs suggests that statin (lipophilic statins) improves surrogate and clinical outcomes in HF. These outcome benefits are attributable to superior pleiotropic effects exhibited by lipophilic statins in HF.^20^, ^21^

Our findings support recent collaborative meta‐analysis of major prevention trials by Preiss et al, which demonstrated significant reduction in risk of HF events (nonfatal HF hospitalization and composite of HF hospitalization and death) with statin therapy in about 132 000 individuals.^52^ This notwithstanding, trials in this meta‐analysis investigated statin therapy in prevention of cardiovascular events including HF, whereas the present study evaluated effects of statins in mortality outcomes of patients with established HF. While Preiss et al did not compare outcomes by statin type, it is plausible that analysis would not identify any differences because of the patient population and mechanisms by which statins reduce events in primary and secondary prevention of cardiovascular events. A potential explanation is that statins may not only reduce the risk of developing HF by preventing ischemic events but also by pleiotropic mechanisms unrelated to LDL‐C reductions. These pleiotropic effects are more pronounced with lipophilic statins compared with hydrophilic statins in patients with HF where the potential mechanism of benefit of statins is believed to be regulation of cardiac inflammation rather than lowering systemic cholesterol levels. Notably, recent meta‐analyses of RCTs have demonstrated that lipophilic statins improve cardiac function and reduce inflammation and show significant reductions in clinical outcomes (mortality and HF hospitalizations) compared with hydrophilic statin treatment in patients with HF.^20^, ^21^

Lipophilic statin treatment was associated with reduced mortality outcomes compared with no statin treatment in our HF cohort. This finding corroborates recent observational studies,^17^, ^18^, ^19^, ^49^ which report overwhelming use of lipophilic statins among patients who received statin treatment in HF. In our cohort, about 80% of patients who were prescribed statin treatment received lipophilic statins (atorvastatin, simvastatin, and fluvastatin). This was evident in the subgroup analyses, which found lipophilic statin use to be associated with significant reduction in mortality outcomes compared with no statin treatment, but this effect was not observed with hydrophilic statin use in patients with HF. Thus, any significant outcome benefits seen with statin use compared with no statin prescription in this study could be attributed to beneficial effects of lipophilic statin in HF.

The observation that lipophilic statins reduce mortality outcomes of patients with HF in our cohort supports the postanalysis of the Treating to New Target study^53^ but in contrast with the 2 large RCTs.^11^, ^12^ The postanalysis of the Treating to New Target study demonstrated that a higher dose of lipophilic statin (atorvastatin) lowers risk of HF hospitalization overall and in particular patients with pre‐existing HF, suggesting a potential direct effect on HF.^53^ These observations lend support to the suggestion that statin treatment affects progression and reduces adverse outcomes of HF despite equivocal outcomes with rosuvastatin treatment in CORONA and GISSI‐HF. Rosuvastatin is hydrophilic, intrinsically hepatoselective, and employs carrier‐mediated mechanisms for uptake into hepatocytes, whereas lipophilic statins enter into cells by passive diffusion and are thus widely distributed in extrahepatic tissues. Moreover, lipophilic statins have very high uptake in myocardial tissue, whereas hydrophilic statins have very low uptake.^54^, ^55^ This is particularly important because a potential mechanism of benefit of statins may be regulation of cardiac inflammation rather than systemic lipid levels in HF. Indeed, lipophilic statins improve cardiac function and reduce inflammation and did show significant reductions in HF hospitalizations, all‐cause and cardiovascular mortality compared with hydrophilic statin (rosuvastatin) treatment in recent meta‐analyses of RCTs involving about 11 000 patients with HF.^20^, ^21^

In the subgroup analyses, hydrophilic statin did not demonstrate any significant reduction in mortality outcomes compared with no statin treatment. In this cohort, the only hydrophilic statin—rosuvastatin—was prescribed for about 18.7% (n=103) of patients who received statins during follow‐up. The lack of outcome benefit seen with rosuvastatin treatment is consistent with the findings of earlier large RCTs and also supports our recent meta‐analysis of statin RCTs in HF. Among the statins, rosuvastatin is the most potent regarding lipid‐lowering effects. This provides backing to suggestions that the potential mechanism underlying the benefit of statins may be more of pleiotropic effects rather than regulating lipid levels in HF. The recent meta‐analysis reported greater treatment effects in reducing inflammation and improving cardiac function with lipophilic statin use compared with hydrophilic statin treatment in HF. Greater pleiotropic effects of lipophilic statins are plausibly responsible for these differences in outcomes compared with hydrophilic statin. While rosuvastatin has shown some pleiotropic properties,^51^ these effects could perhaps be inadequate to produce significant outcome benefits in patients with HF. Nonetheless, the lack of outcome benefit with rosuvastatin could also be attributed to the small sample of patients who received hydrophilic statins during follow‐up. The sample size could have resulted in frail estimates because of insufficient power to test for any differences in outcomes between treated and untreated groups.

Besides the uncertainty about generalizability of earlier large trials because of the focus on hydrophilic statin (rosuvastatin), an important point worth considering is the fact that the patient groups were overwhelmingly of white background. Earlier studies have suggested varied responses to approved HF treatments between patients of African descent and whites.^26^, ^27^, ^28^, ^29^, ^56^, ^57^ Our cohort of black Africans may be related to the black American population because of comparable cardiovascular risk factors^23^, ^24^, ^58^ and similar age of HF onset. In this patient population, the efficacy of mainstay treatments for HF is unclear.^27^, ^28^, ^29^ Moreover, they are among the ethnic minorities, who are underrepresented in major RCTs.^49^, ^59^ Some have suggested that rosuvastatin could have failed to show significant reduction in primary end points or had attenuated treatment effects because patients enrolled in the large trials were already receiving optimal regimens of approved HF treatments. On the contrary, the present study was conducted in a population in which efficacies of mainstay HF treatments remain indistinct and thus significant reductions in mortality outcomes observed with statin treatment could plausibly be attributable to the true effects of statins in HF. While there are no reasons to suspect that statins will not produce similar outcomes in Africans compared with whites with HF, it was important to verify this assumption. Furthermore, the need to reduce the poor prognosis, paucity of data from controlled trials of HF treatments, as well as the lack of clarity surrounding the efficacy of approved HF treatments are strong indications for assessing the effects of statin therapy in black Africans with HF. Thus, in the absence of any subgroup analysis of CORONA and GISSI‐HF data targeting racial differences in response to statins, a well‐conducted observational study such as the present study in a predominantly black population will help provide considerable evidence and recommendations for statin use. Also, this evidence for statin treatment added to standard therapy among black patients with HF may provide a conundrum for clinicians and researchers in interpreting the absence of benefit in the 2 large RCTs.

### Strength and Limitations

This study possesses several unique strengths that require comment. First, to the best of our knowledge, this is the first study to evaluate the effect of statins in a predominantly black African population with HF.

Second, unlike previous observational studies evaluating statin treatment in HF, we employed a new user approach to our study cohort of newly diagnosed HF. Specifically we excluded patients with statin exposure 3 months before date of index admission for HF, overcoming any potential issues with an attenuated effect. The new user approach addresses bias that could be introduced by inclusion of prevalent statin users.^32^ Third, our cohort, with a considerable prevalence of comorbidities and prescribed co‐medications, adequately represents the patient population in real‐world clinic settings. Next, the present study has data on some important clinical factors such as left ventricular ejection fraction and LDL‐C, which are essential to determining treatment effects of statins in HF throughout the 5‐year follow‐up. Finally, concern about nonrandomized treatment allocation in an observational study was addressed by creating a pseudorandomized sample by applying IPTWs to the sample.^33^, ^35^ As the time‐dependent Cox model cannot address bias estimation of treatment effect associated with time‐varying confounding by indication, we further carried out the analysis using MSM. Thus, our results were robust independent of the analytical strategy and the subgroup analyses performed.

The interpretation of results of this study should be made in light of several limitations. First, in the hierarchy of strengths of evidence, RCTs are superior to observational studies like the present study because of confounding. Although our study suggests a relationship between statin therapy and improved clinical outcomes, it was observational in nature, and therefore we cannot definitively infer a causal relationship as might be anticipated from a RCT of statin efficacy. Second, for causal associations determined by MSM estimates to be valid, we make an assumption of no unmeasured confounding, which cannot be tested. However, it is possible any potential unmeasured confounders would be somewhat correlated with the numerous sociodemographic, clinical, and treatment factors that were measured, as a consequence reducing residual confounding. Third, dispensed prescriptions were considered as actually consumed. However, in general, pharmacy claims are demonstrated to be an accurate measure of prescription drug consumption.^34^ To the extent possible, measures of adherence to statin prescribed were clearly defined in study protocol to strengthen data quality and analyses. Despite known limitations, the modified Framingham criterion is 100% sensitive and 78% specific in identifying patients with HF.^30^ To minimize inaccuracies in diagnosis using this criterion, we excluded patients without left ventricular ejection fraction data from the analysis. In the present study, the diagnosis of HF was based on the modified Framingham criteria and supported with echocardiographic data. Although tissue Doppler imaging was not performed, this did not affect the diagnosis of HF because clinical assessment was mainly based on left ventricular ejection fraction and modified Framingham criteria. Finally, while data for this study come from a reputable institution with a cardiac clinic, data sources are paper based, and we cannot completely rule out inaccuracies in entries, particularly regarding the cause‐specific mortality. To the extent possible, we attempted to solve any disagreements by consensus between researchers and clinicians during data abstraction.

## Conclusions

In the absence of adequately powered RCTs, appropriate adjustment for time‐varying confounding by indication may provide the best evidence to estimate treatment effects in observational studies. This exploratory study using time‐dependent Cox model and MSM found significant association between statin treatment and all‐cause mortality, cardiovascular mortality, and HF mortality among Africans with newly diagnosed HF. Furthermore, lipophilic statin treatment but not hydrophilic statin was associated with significantly reduced mortality outcomes in HF. Our findings suggest that additional sufficiently powered randomized trials evaluating statins other than hydrophilic rosuvastatin with longer follow‐up period will be necessary. Also, it would be interesting to compare the effects of lipophilic versus hydrophilic statin treatment in a head‐to‐head trial in patients with HF.

## Author Contributions

Bonsu, Kadirvelu, and Reidpath conceived the study idea and design. Bonsu, Owusu, and Buabeng provided administrative, technical, or material support for the study. All authors were responsible for the study and acquisition of data. Bonsu, Owusu, and Reidpath had full access to the data in the study and take responsibility for the integrity of the data and the accuracy of the data analysis. Bonsu and Reidpath analyzed the data. All authors contributed to the interpretation of data. Bonsu drafted the initial manuscript and all authors contributed to subsequent drafts.

## Disclosures

None.
